# Profile of Sensory Integration Disorders in Migraine Patients—New Perspectives of Therapy

**DOI:** 10.3390/jcm13133928

**Published:** 2024-07-04

**Authors:** Agata Kaniewska, Ewelina Bagińska, Marta Masztalewicz, Krystian Mross, Marta Jankowska, Przemysław Nowacki, Agnieszka Meller, Karolina Machowska-Sempruch, Wioletta Pawlukowska

**Affiliations:** 1 Department of Neurology, Pomeranian Medical University, Unii Lubelskiej 1, 71-252 Szczecin, Poland; marta.masztalewicz@pum.edu.pl (M.M.); przemyslaw.nowacki@pum.edu.pl (P.N.); agnieszka.meller@pum.edu.pl (A.M.); karolina.machowska.sempruch@pum.edu.pl (K.M.-S.); wioletta.pawlukowska@pum.edu.pl (W.P.); 2 Doctoral School, Pomeranian Medical University, 71-210 Szczecin, Poland; 34650@student.pum.edu.pl (E.B.);

**Keywords:** sensory processing disturbance, headache, aura, hypersensitivity, hyposensitivity

## Abstract

**Background:** The involvement of sensory integration disorders in the pathophysiology of migraine has been suggested. This study aims to analyze the relationship between symptoms of sensory integration disorders and migraine in a broad scope, including all sensory domains, and examine its impact on migraine attacks. **Methods**: The study included 372 people diagnosed with migraine. The Daniel Travis Questionnaire was used to assess symptoms of sensory integration disorders and their severity across six domains. The relationships between the severity of these symptoms and headache features, as well as accompanying headache symptoms, were the subject of statistical analysis. **Results:** Current impairment in all sensory domains was significantly associated with headaches exacerbated by everyday life activities. A significant inverse relationship was found between the occurrence of throbbing headaches and symptoms of sensory integration disorders in terms of current sensory discrimination, current motor skills, and current emotional/social skills. Past under-responsiveness and past disturbances in emotional/social abilities were significantly associated with migraine aura. **Conclusions:** The severity of symptoms of sensory integration disorders affects the clinical picture of migraine. The significant association between migraine and emotional/social disorders, as well as under-responsiveness in the past, needs further research to assess whether this is a cause-and-effect relationship. There is a need for in-depth diagnostics of sensory integration disorders in migraine patients, which could be an additional target of their therapy.

## 1. Introduction

Migraine is a headache disorder manifested by recurrent 4–72 h headache attacks that have at least two of the following characteristics: unilateral location, pulsating quality, moderate to severe pain intensity, and aggravation during activities of daily living (ADL) [1]. Additionally, at least one of the following symptoms is present during attacks: nausea and/or vomiting, photophobia, and phonophobia [1]. Migraine might be preceded by an aura, as well as prodromal symptoms [1,2].

Epidemiological studies show that migraine is the second leading cause of disability worldwide and first among young women [3]. The migraine patients experience a significant reduction in quality of life (QoL), affecting both professional and social aspects of daily functioning [4]. Furthermore, it has been proven that the reduction of health-related quality of life (HRQoL) in this disease depends on the severity and frequency of migraine attacks. The reduction in HRQoL is present, both during migraine attacks and interictal periods [5].

Despite the introduction of new drugs into therapy, there is still a paucity of migraine treatment efficacy [6,7,8]. Research into the pathogenesis of migraine has resulted in a better understanding of the structures and mechanisms responsible for the onset and recurrence of migraine pain [9]. Therefore, it appears that pharmacotherapy is not the only or complete way to improve the lives of migraine patients. Focusing on sensory integration disorders (SID), also known as sensory processing disorder (SPD), may be a useful element of the therapy of the patients. The essence of somatosensory integration disorders is the incorrect integration of visual, auditory, tactile, olfactory, gustatory, vestibular, and proprioceptive stimuli, which affects the adopted behavioral patterns [10,11]. People may have a reduced or increased threshold for receiving certain types of stimuli, which generates a subsequent pattern of behavior in contact with given stimuli. This results in subjects exhibiting exaggerated responses to sensation in a hyper- or hyporesponsive manner, as well as sensory-seeking behaviors, poor sensory discrimination, and poor sensory-based motor abilities [12]. The diagnosis of SPD should include an analysis of the patient’s functioning in each of these domains [13,14,15]. There is no standardized way to evaluate sensory processing in adults. The assessment is based on an interview with patients, to which questionnaires assessing the severity and frequency of symptoms of disorders can be used. The Daniel Travis Questionnaire, which assesses all domains of somatosensory integration disorders, is such a questionnaire [16].

The involvement of somatosensory integration disorders in the pathophysiology of migraine has been suggested [17,18,19]. Drawing attention to somatosensory integration disorders in migraine stems from the fact that migraine is accompanied by the disorders of multisensory perception—visual, auditory, olfactory, and tactile. Hypersensitivity to light, sound, and smell, as well as painful sensation of touch, is a strong part of the clinical picture of migraine—during and between migraine attacks [20,21]. Current research focuses mainly on over-responsivity [22]. It is not known what the disorders in other domains are in this group, as well as whether and how the sensory processing disorders in question affect the clinical picture of migraine. This is a topic worth attention from the point of view of searching for additional therapies in this group of patients.

This study aims to analyze the occurrence and severity of impairment in all domains of Somatosensory Processing Disorder in people with migraine. In the next step, it analyzes all sensory domains in relation to the characteristics of migraine attacks.

It is hypothesized that sensory processing disorder in patients with migraine involves more than one domain. The profile of these disorders may be an important piece in the clinical picture of migraine.

## 2. Materials and Methods

This is a cross-sectional study.

### 2.1. Material

The participants were people with migraines, and they completed a self-administered questionnaire shared through the website from 1st March 2023 to 31st July 2023. The study involved 372 (359 women and 13 men; mean age ± SD: 34.7 ± 9.4) out of 400 people who completed the questionnaire and, at the same time, met the criteria for participation in the study. Data collection was closed after this time due to the lack of further activity in completing the questionnaire, and the number of people that could be recruited far exceeded the estimated minimum group size needed for the planned analyses.

The sample size was estimated at 267 persons at a significance level of 0.05, a general population size of 3,200,000 persons, with an acceptable error of 6%. The inclusion criteria were participants’ declaration that migraine diagnosis was established by the neurologist, and the diagnosis was positively verified according to ICHD-3rd (the International Classification of Headache Disorders, third edition) criteria for migraine without aura and migraine with aura, i.e., experiencing a minimum of five pain attacks lasting 4–72 h if not preceded by the aura or at least two attacks if preceded by the aura. Among headache features, a minimum of two of the following characteristics are needed: unilateral pain, throbbing pain, moderate to severe severity, and avoidance of activities of daily living. In addition, a minimum of one additional symptom is present during an attack: nausea and/or vomiting, phonophobia, and photophobia. The symptoms reported by the patients should not correspond to another diagnosis according to ICHD-3 [1]. The 28 participants mentioned above were people who did not report that migraine diagnosis was established by a neurologist. Some of them declared that migraine diagnosis was established by a neurologist, but they did not meet the diagnostic criteria for migraine according to ICHD-3 based on our verification.

### 2.2. Methods

A self-administered questionnaire, shared through the website, was used to obtain data about age, sex, and education level. They were asked about headache features, like unilateral, pulsating pain, pain intensity according to Numeric Rating Scale (moderate pain assessed 5–6 points and severe pain 7–10), pain aggravated during normal activity/causing avoidance of daily activity, and headache duration. They were asked about additional symptoms, like sensitivity to light, to noise, and nausea/vomiting.

The above-mentioned information about headache features and accompanying symptoms was used to confirm that the person has migraine-like headaches [1].

The other question was how many episodes of migraine-like headaches the patient had up to the time he/she filled out the questionnaire. More than four were considered to confirm the diagnosis [1]. Any comorbidities were also considered in this regard [1].

Other questions were about the frequency of migraine attacks (average number of attacks per month, considering at least the last three months), the occurrence of non-migraine headaches, the duration of the disorder, taking medications, including medications for migraine (as abortive and preventive treatment).

This study utilizes the Daniel Travis Questionnaire, following the guidelines of the American Society SPD in Adults, to assess symptoms of SPD in individuals aged 18 and older. This collection of symptoms indicating sensory integration disorder is part of the patient’s medical history. An increased frequency or high severity of symptoms may suggest a sensory integration disorder; however, further evaluation is needed.

The Daniel Travis questionnaire was previously used on the Polish population with the supervision of a certified sensory integration therapist. In our study, one of the authors is a certified Ayres Sensory Integration Therapist and Sensorisch-Integrative Ayres Therapie [16,23]. It distinguishes the domains of general modulation (GM, 9 statements), over-responsiveness (OR, 26 statements), under-responsiveness/sensory seeking (UR/SS, 20 statements), sensory discrimination (DS, 26 statements), sensory-based motor abilities (SBMA, 19 statements) and social and emotional (SE, 22 statements). Each symptom was rated by the study participant using a 5-point Likert scale from 0 (never occurred) to 4 (regularly occurs). If the problem had occurred in the past but no longer persists, the participant entered the letter P. The sum obtained by adding the values in each domain indicated the severity of the SPD disorder in that domain.

### 2.3. Statistical Analysis

Statistical analysis was performed using the licensed Statistica 13.3 program (StatSoft, Inc., Tulsa, OK, USA). Means, medians, and, respectively, standard deviations, lower and upper quartiles, as well as numbers and percentages, were used to present the characteristics of the group. The normality of the distribution of the studied variables was assessed using the Shapiro–Wilk test. The analysis of homogeneity of variance was performed using Levene’s test. In almost all analyses, non-normal distribution or heterogeneity of variance was obtained, therefore non-parametric tests were used for further analysis of quantitative data. For statistical analysis, the group of patients with migraine was divided into subgroups based on the presence–absence of particular migraine symptoms: pain caused/exacerbated by activities of daily living, unilateral pain, pulsating pain, the pain of moderate to severe intensity, headache preceded by aura, nausea/vomiting, and photo-/phonophobia. The analysis between these subgroups was performed using the Mann–Whitney U test due to unequal variance. The dependent variables were analyzed using the Wilcoxon matched-pairs test. The significance level of *p* < 0.05 was assumed (Table 1).

## 3. Results

Comparing the severity of current and past sensory integration disorders in the analyzed group, we have observed that they are significantly more severe currently than in the past. It concerned each domain (Table 2).

General modulation, over-responsiveness and sensory discrimination, sensory-based motor abilities, as well as social and emotional skills currently were significantly more disturbed among patients with headaches aggravated by ADL compared to patients without this headache feature (Table 3). Under-responsiveness/sensory seeking currently, as well as in the past, was also significantly associated with sensitivity to ADL (Table 3).

We did not find any significant association between particular SPD domains and unilateral character of headache (Table 4).

A significant inverse association was found between the occurrence of throbbing headaches and sensory integration disorders in terms of current sensory discrimination, current motor skills, and current emotional/social skills (Table 5).

We did not find any significant association between particular SPD domains and moderate to severe character of headache (Table 6).

The severity of current general modulation disturbance, over-responsiveness, under-responsiveness/sensory seeking, and social and emotional abilities disturbance were significantly associated with hypersensitivity to light and/or noise during migraine attacks (Table 7).

There were no associations between SPD domains (currently, as well as in the past) and nausea/vomiting occurrence (Table 8).

Under-responsiveness in the past and social-emotional abilities disturbance in the past were significantly more severe in patients experiencing headaches preceded by aura (Table 9).

## 4. Discussion

Research indicates the influence of abnormal activation of the cerebral cortex in the pathogenesis of migraine, with the involvement of the structures of the somatosensory system in close relationship with the trigeminovascular system [17,18,24]. The occurrence of sensory integration disorders in migraine patients is manifested by cutaneous allodynia, photophobia, phonophobia, and osmophobia. These are clinical symptoms accompanying most migraine attacks [19,20,21]. These ailments may be caused by increased sensitivity, i.e., one of the sensory domains, which, in turn, may influence the overmodulation of other domains.

Our study has shown that people with migraine not only have increased sensitivity but also show significant impairments in all somatosensory domains. Considering these results, it is reasonable to conclude that migraine may be associated with sensory integration disorders.

In the analyzed population of migraine patients, a significant association was found between disorders of each sensory domain and sensitivity to everyday activities. These disorders were significantly more severe in people who experienced headaches worsened by ordinary activities or avoided performing any activities during a migraine attack. During an attack, a patient with this feature of migraine needs silence, peace, as little movement as possible, and as little activation of the senses as possible [25]. This condition appears to reflect chaos in cortical excitation and inhibition, with a consequence of altered multisensory processing [17,26].

Increased sensitivity to everyday activities was also associated with sensory hyposensitivity in the past. Bayes created a theory of learning and showed that disruption of one of the sensory domains in developmental age can lead to “sensory uncertainty”, which translates into a shortening of the time of the presented stimulus [27,28,29,30]. The sensory chaos may evolve over time. The influence of sensory disruption in the past on current sensory domains has been demonstrated, leading to impaired learning and stimulus adaptation [27]. Considering our study, a history of sensory hypersensitivity disorders may lead to dysregulation of all sensory domains in people with migraine.

In almost two-thirds of the analyzed patients, the headache pain was throbbing. It was found that these people had significantly fewer somatosensory integration disorders in terms of sensory discrimination, motor skills, and emotional/social skills compared to patients without throbbing headaches. Research conducted on children has shown that motor functions, emotional abilities, and sensory discrimination constitute a closed entity interacting with each other. Sensory feedback plays an important role in learning new motor skills. Peripheral sensory stimuli increase the excitability of the motor cortex [31]. Studies conducted in patients with Parkinson’s disease have shown that movement disorders are dependent on sensory discrimination disorders [32]. Motor performance is also related to emotional processing [33]. Interestingly, emotional modulations observed both at the early stage of structural development and the later stage of attentional engagement may be the result of interactions between movement and emotion-related information conveyed by emotional body postures. The body expresses emotions through movements, thereby providing simultaneous movement-related information. Therefore, movement and sensory and emotional discrimination functions are self-driving distractors [34].

These associations may indicate the involvement of the dysfunction of these three distractors in the occurrence of throbbing headaches in the migraine patients analyzed in this study. Migraine headaches are worsened by physical activity. During a migraine attack, the perivascular endings of the trigeminal nerve are activated, which leads to the release of pro-inflammatory substances, vasoconstrictors, and vasodilators. As a result, the tissue surrounding the arteries, especially in the meninges, becomes sensitized. Thus, normal pulsations that are not even felt under normal conditions may be felt as pain during a migraine attack. In this setting, any activity that increases heart rate and/or arterial flow causes increased pulsations, which patients experience as throbbing pain (sport and exercise-induced migraines). Studies have shown that physical activity worsens throbbing headaches but reduces the frequency of attacks. Most likely, people with reduced motor skills avoid sports, which reduces the occurrence of throbbing headaches that are provoked by physical exercise [35,36].

During a migraine attack, 80–90% of patients experience photophobia and 70–80% experience phonophobia. Photophobia and phonophobia are part of the diagnostic criteria for migraine. Additionally, migraine patients often experience photophobia, and phonophobia between attacks, and have a lower discomfort threshold in response to visual, auditory, and tactile stimuli in the interictal period compared to non-migraine controls [20,21].

In our group of patients, almost 93% of people with migraine had photo and phonophobia. We showed that these symptoms occurred in people with sensory hyper- or hyposensitivity and emotional disorders. According to the research, emotional disorders result from sensory hyposensitivity. The fMRI studies have shown that migraine sufferers show greater reactivity to visual stimuli in the visual cortex and visual association areas, and it is thought that brain hyperactivity may be associated with symptoms of photophobia. Interictal photosensitivity in migraine patients is associated with structural brain aberrations, including greater cortical thickness in the left frontoparietal and right parieto-occipital regions. Migraine patients also show increased interictal connectivity between the auditory and visual cortices and the anterior insula, which is involved in emotional clarity [37]. Hence, emotional disorders occur in these people. Another form of sensory hypersensitivity is probably related to hypersensitivity of pain processing and modulation areas, including first- and second-order trigeminovascular neurons and thalamic nociceptive neurons [38].

Our study showed that sensory under-responsiveness and over-responsiveness to light and odors are strongly associated. It is worth checking what kind of association this is. Some studies have shown that olfactory functioning is also impaired in sensory hyposensitivity [39].

Migraine with aura occurred in over 30% of the analyzed population. This feature of migraine is closely related to impaired cerebral cortex function (cortical spreading depression) [24]. Past sensory hypersensitivity disorders and emotional disturbances have been observed to be strongly associated with migraine with aura. Sensory integration disorders can be treated. Therefore, it should be investigated whether the inclusion of sensory integration therapy in people with migraine will have an impact on the course of the disease.

According to the literature data, depression that begins in adolescence is associated with a high risk of recurrence in adulthood. Moreover, emotional disorders in childhood increase the occurrence of increased stress and anxiety in adulthood. Sensory hyposensitivity is associated with abnormal emotional behavior, search for sensory impressions, and impulsivity. People with a history of emotional disorders react to sudden stress with panic and anxiety [27,28]. Can providing such contradictory information to the central nervous system lead to “information conflict”, which translates into the appearance of an aura? This hypothesis requires better testing, but considering the mechanisms occurring in migraine, it is highly probable.

The collected database shows a certain sensory integration profile characteristic of people suffering from migraines: people who need self-stimulation, we can call them sensory seekers, and those who are over-responsive, sensory avoiders. The goal of sensory integration therapy for this group of patients would be to improve sensory processing of the nervous system and consequently influence migraine symptoms.

### Study Limitations

Our study is a prelude to further research into the association of migraine with SPD occurrence. It is certain that the disorder encompasses all domains and not only the occurrence of hypersensitivity, as thought until now. While we have extracted a specific sensory integration profile specific to people with migraine (based on the assessment of the presence of main SPD symptoms), it is worthwhile to deepen this topic based on the full SPD diagnosis. A weakness of this study is also the fact that it was survey-based.

Separate research on the significance of SPD in the pathophysiology of migraine is needed.

## 5. Conclusions

1. Somatosensory integration disorders in migraine concern hypersensitivity to stimuli, as well as affect other domains.
2. The scope of SPD affects the clinical picture of migraine, especially the occurrence of sensitivity to everyday activities, the pulsating nature of the headache, photophobia, and hypersensitivity to noise.
3. The significant relationship between migraine and emotional disorders, as well as under-responsiveness in the past, needs further research to assess whether this is a cause-and-effect relationship.
4. Based on the frequency of SPD symptoms in migraine patients, there are strong reasons for in-depth diagnostics of sensory integration disorders in these patients, which could be an additional target of therapy.

## Figures and Tables

**Table 1:** The characteristics of analyzed migraine patients (*n* = 372).

Demographic Data		
34.7 ± 9.4	Age (Years; Mean ± SD)	
359 (96.50%)	Females	Sex, *n* (%)
13 (3.49%)	Males	
0 (0.00%)	Primary	Education, *n* (%)
9 (2.42%)	Vocational	
92 (24.73%)	High school	
271 (72.85%)	University	
Migraine features		
242 (65.05%)	Yes	Pain caused/exacerbated by activities of daily living, *n* (%)
130 (34.95%)	No	
306 (82.26%)	Yes	Unilateral pain, *n* (%)
66 (17.74%)	No	
236 (63.44%)	Yes	Pulsating pain, *n* (%)
136 (36.56%)	No	
233 (62.73%)	Yes	Pain of moderate intensity, *n* (%)
139 (37.26%)	No	
234 (62.90%)	Yes	Headache preceded by aura, *n* (%)
138 (37.09%)	No	
281 (75.54%)	Yes	Nausea/vomiting, *n* (%)
91 (24.46%)	No	
347 (93.28%)	Yes	Photo-/phonophobia, *n* (%)
25 (6.72%)	No	

**Table 2:** Current and past distribution of Sensory Processing Disorder domains in migraine patients using Daniel Travis Questionnaire.

*p*	Disturbance Intensity	Sensory Processing Disorder Domain
0.000	13.502 ± 7.534	General modulation
	0.061 ± 0.241	General modulation in the past
0.000	40.693 ± 20.813	Over-responsiveness
	0.247 ± 0.702	Over-responsiveness in the past
0.000	27.115 ± 15.395	Under-responsiveness/sensory seeking
	0.451 ± 1.425	Under-responsiveness/sensory seeking in the past
0.000	25.879 ± 18.326	Sensory discrimination
	0.494 ± 1.283	Sensory discrimination in the past
0.000	18.989 ± 15.048	Sensory-based motor abilities
	0.263 ± 1.195	Sensory-based motor abilities in the past
0.000	36.387 ± 18.391	Social and emotional
	0.432 ± 1.539	Social and emotional in the past

**Table 3:** Sensory Processing Disorder domains in migraine patients using Daniel Travis Questionnaire in relation to headache features: pain caused/exacerbated by activities of daily living—assessed with ICHD-3 criteria.

Sensory Processing Disorder Domain	Pain Caused/Exacerbated by Activities of Daily Living		*p*
	Yes	No	
	(*n* = 242)	(*n* = 130)	
General modulation	14.566	11.523	0.000
General modulation in the past	0.239	0.261	0.711
Over-responsiveness	42.462	37.400	0.015
Over-responsiveness in the past	0.512	0.338	0.251
Under-responsiveness/sensory seeking	28.747	24.076	0.002
Under-responsiveness/sensory seeking in the past	0.566	0.361	0.023
Sensory discrimination	27.132	23.546	0.034
Sensory discrimination in the past	0.268	0.253	0.406
Sensory-based motor abilities	16.769	15.421	0.027
Sensory-based motor abilities in the past	0.210	0.215	0.630
Social and emotional	39.016	31.492	0.000
Social and emotional in the past	0.396	0.500	0.867

**Table 4:** Sensory Processing Disorder domains in migraine patients using Daniel Travis Questionnaire in relation to headache features: unilateral pain—assessed with ICHD-3 criteria.

Sensory Processing Disorder Domain	Unilateral Pain		*p*
	Yes	No	
	(*n* = 306)	(*n* = 66)	
General modulation	13.075	13.594	0.589
General modulation in the past	0.212	1.022	0.274
Over-responsiveness	41.026	21.355	0.589
Over-responsiveness in the past	0.651	0.408	0.672
Under-responsiveness/sensory seeking	26.166	27.320	0.403
Under-responsiveness/sensory seeking in the past	0.545	0.483	0.999
Sensory discrimination	26.090	25.833	0.787
Sensory discrimination in the past	0.469	0.218	0.484
Sensory-based motor abilities	17.393	19.333	0.248
Sensory-based motor abilities in the past	0.363	0.179	0.559
Social and emotional	35.924	36.486	0.930
Social and emotional in the past	0.651	0.385	0.929

**Table 5:** SPD domains in migraine patients using Daniel Travis Questionnaire in relation to headache features: pulsating pain—assessed with ICHD-3 criteria.

Sensory Processing Disorder Domain	Pulsating Pain		*p*
	Yes	No	
	(*n* = 236)	(*n* = 136)	
General modulation	12.897	13.851	0.280
General modulation in the past	0.235	0.254	0.949
Over-responsiveness	39.220	41.542	0.293
Over-responsiveness in the past	0.455	0.449	0.415
Under-responsiveness/sensory seeking	25.485	28.055	0.113
Under-responsiveness/sensory seeking in the past	0.544	0.466	0.510
Sensory discrimination	23.183	27.432	0.038
Sensory discrimination in the past	0.360	0.207	0.876
Sensory-based motor abilities	16.536	20.402	0.010
Sensory-based motor abilities in the past	0.205	0.216	0.655
Social and emotional	33.786	37.885	0.037
Social and emotional in the past	0.286	0.516	0.244

**Table 6:** Sensory Processing Disorder domains in migraine patients using Daniel Travis Questionnaire in relation to headache features: moderate to severe pain—assessed with ICHD-3 criteria.

Sensory Processing Disorder Domain	Moderate to Severe Pain		*p*
	Yes	No	
	(*n* = 233)	(*n* = 139)	
General modulation	13.678	13.208	0.617
General modulation in the past	0.227	0.280	0.664
Over-responsiveness	40.716	20.728	0.813
Over-responsiveness in the past	0.351	0.618	0.299
Under-responsiveness/sensory seeking	26.583	28.007	0.358
Under-responsiveness/sensory seeking in the past	0.480	0.517	0.239
Sensory discrimination	24.836	27.625	0.219
Sensory discrimination in the past	0.150	0.453	0.103
Sensory-based motor abilities	19.064	18.863	0.850
Sensory-based motor abilities in the past	0.154	0.309	0.351
Social and emotional	36.858	35.597	0.435
Social and emotional in the past	0.381	0.517	0.596

**Table 7:** Sensory Processing Disorder domains in migraine patients using Daniel Travis Questionnaire in relation to migraine accompanying symptoms: hypersensitivity to noise, light—assessed with ICHD-3 criteria.

Sensory Processing Disorder Domain	Hypersensitivity to Noise, Light		*p*
	Yes	No	
	(*n* = 347)	(*n* = 25)	
General modulation	13.876	8.320	0.000
General modulation in the past	0.253	0.160	0.934
Over-responsiveness	41.590	28.240	0.001
Over-responsiveness in the past	0.435	0.680	0.355
Under-responsiveness/sensory seeking	27.766	18.080	0.000
Under-responsiveness/sensory seeking in the past	0.498	0.440	0.475
Sensory discrimination	26.449	17.960	0.008
Sensory discrimination in the past	0.259	0.320	0.927
Sensory-based motor abilities	19.265	15.160	0.309
Sensory-based motor abilities in the past	0.201	0.360	0.318
Social and emotional	37.074	26.840	0.008
Social and emotional in the past	0.452	0.160	0.536

**Table 8:** Sensory Processing Disorder domains in migraine patients using Daniel Travis questionnaire in relation to migraine accompanying symptoms: nausea or vomiting—assessed with ICHD-3 criteria.

Sensory Processing Disorder Domain	Nausea, Vomiting		*p*
	Yes	No	
	(*n* = 281)	(*n* = 91)	
General modulation	13.351	13.551	0.934
General modulation in the past	0.274	0.238	0.908
Over-responsiveness	39.857	40.964	0.881
Over-responsiveness in the past	0.494	0.437	0.448
Under-responsiveness/sensory seeking	28.219	26.758	0.393
Under-responsiveness/sensory seeking in the past	0.395	0.526	0.191
Sensory discrimination	27.021	25.508	0.259
Sensory discrimination in the past	0.186	0.288	0.432
Sensory-based motor abilities	17.461	19.483	0.518
Sensory-based motor abilities in the past	0.230	0.206	0.583
Social and emotional	38.109	35.829	0.230
Social and emotional in the past	0.461	0.423	0.231

**Table 9:** Sensory Processing Disorder domains in migraine patients using Daniel Travis Questionnaire in relation to migraine accompanying symptoms: headache preceded by an aura—assessed with ICHD-3 criteria.

Sensory Processing Disorder Domain	Headache Preceded by an Aura		*p*
	Yes	No	
	(*n* = 234)	(*n* = 138)	
General modulation	13.442	13.538	0.837
General modulation in the past	0.239	0.252	0.801
Over-responsiveness	40.847	40.602	0.901
Over-responsiveness in the past	0.384	0.491	0.660
Under-responsiveness/sensory seeking	25.833	27.871	0.354
Under-responsiveness/sensory seeking in the past	0.420	0.538	0.047
Sensory discrimination	25.137	26.316	0.508
Sensory discrimination in the past	0.275	0.256	0.953
Sensory-based motor abilities	18.833	19.081	0.793
Sensory-based motor abilities in the past	0.188	0.226	0.405
Social and emotional	36.847	36.115	0.793
Social and emotional in the past	0.231	0.551	0.016

## Data Availability

The data cannot be made publicly available due to privacy regulations.
